# Epidemiology and associated injuries in paediatric diaphyseal femur fractures treated at a limited resource zonal referral hospital in northern Tanzania

**DOI:** 10.1186/s12891-022-05320-x

**Published:** 2022-04-18

**Authors:** Albert P. Macha, Rogers Temu, Frank Olotu, Neil P. Seth, Honest L. Massawe

**Affiliations:** 1 Department of Orthopaedic and Trauma, Muhimbili Orthopaedic Institute, P.O.Box 38645, Dar es Salaam, Tanzania; 2 Department of Orthopaedic Surgery, Kilimanjaro Christian Medical University College, Moshi, Kilimanjaro Tanzania; 3 Department of Orthopaedic Surgery, Kilimanjaro Christian Medical Centre, Moshi, Kilimanjaro Tanzania; 4 Department of Orthopaedic Surgery, Pennsylvania Hospital, University of Pennsylvania, Philadelphia, USA

**Keywords:** Orthopaedic Injuries, Childhood, Fracture, Femur

## Abstract

**Background:**

Diaphyseal femur fractures contribute up to 40% of paediatric orthopaedic admissions with the World Health Organisation data showing youth are particularly vulnerable and road traffic injuries are the leading cause of death for children and young adults. Different mechanisms results to these injuries and they vary with age and geographical location of the patient. Understanding the incidence, mechanism and pattern of these injuries allows planning for preventive measures and treatment to meet modern day patient demands, generation of appropriate and timely protocols with minimum social and economic burden to the patient and family.

**Objectives and methods:**

A hospital based cross sectional study was conducted using the orthopaedic department patient registry among children aged under 18 years admitted from 2014—2018. Our research question was to determine the epidemiology of diaphyseal femur fractures and coexisting associated injuries among admitted paediatric orthopaedic patients. Patient files were reviewed from the medical records department and a data collecting sheet was used to record demographics and injury data. Odds ratios with 95% confidence intervals for associated injuries in paediatric diaphyseal femur fractures were estimated using multivariable logistic regression model.

**Results:**

We found the prevalence of diaphyseal femur fracture among paediatric orthopaedic admissions was 18% with the majority 111 (68.5%) being males. The leading injury mechanism was a fall (57.4%) followed by road traffic injuries (35.8%) out of which 48.3% resulted from pedestrian vs motorcycle accidents. Traumatic brain injury (TBI) was the most common associated injuries accounting for 69% of these injuries with the majority 79% occurring in patients aged 6 years and older. With age specific analysis, children in 6–12 years and 13–18 years age groups, had 8 and 11 times higher odds for associated injuries (OR 8.25, 95% CI, 1.04—65.31) *p* = 0.046 and (OR 10.54, 95% CI, 1.26—88.31) *p* = 0.031 respectively compared to those younger ≤ 2 years. Road traffic related injuries had 17 times higher odds of associated injuries when compared to fall (OR 16.73, 95% CI, 6.28—44.57) *p* < 0.001. 112 (69.1%) of femur fractures were treated by non-operative method out of this 90 (55.6%) by traction with delayed Spica application. The overall mean duration of hospital stay was 18.5 ± 11 days.

**Conclusion:**

Pedestrian vs motorcycle injuries was the leading specific cause of paediatric diaphyseal femur fractures with TBI being the common associated injury. Non-operative management was the most utilized treatment plan and contributed to ten times higher odds for a longer duration of hospital stay. Initiatives to insure children safety on roads should be strengthened in order to reduce/eliminate this burden. Application and practice of current evidence based clinical guidelines and recommendations is paramount for timely and appropriate treatment of these injuries.

**Supplementary Information:**

The online version contains supplementary material available at 10.1186/s12891-022-05320-x.

## Introduction

The World Health Organization (WHO) endorse injury as the leading cause of morbidity and mortality in children after their first year of life [1]. Diaphyseal femur fractures account for up to 40% of paediatric orthopaedic admissions in low and middle income countries [2]. The mechanism of injury for these fractures vary with patient age; falls from standing height or playground equipment are more in younger children while high energy injuries are more commonly seen in adolescents and older children [2, 3]. Recent studies report the significant rise in road traffic injuries (RTI) as the major cause of these fractures [3–5].

Associated bodily injuries are common in children that sustain a diaphyseal femur fracture. High energy injury mechanisms increase the risk of associated injuries; and the presence of an associated injury portends a two-fold increase in the hospital length of stay (LOS) irrespective of the treatment modality employed [6–8]. Understanding the mechanism of injury and the resulting pattern of paediatric femur fractures is essential to determine the risk of associated injuries among children. Currently, limited information is available regarding the magnitude and trends of this clinical scenario in sub-Saharan Africa.

We conducted a retrospective audit over five years to determine the mechanism of injury of paediatric diaphyseal femur fractures and associated injuries at a zonal referral-teaching hospital in northern Tanzania. Our research question was to determine the prevalence of diaphyseal femur fractures and coexisting associated injuries among admitted paediatric orthopaedic patients. Defining the magnitude of this problem will be a platform for planning and initiating preventive measures [9–11].

## Material and methods

The institutional review Ethical and Research Committee approved the study with certificate number 2333. We performed a retrospective, cross-sectional study of all paediatric orthopaedic patients with diaphyseal femur fractures from January 2014 to December 2018 admitted to the Orthopaedic ward at a zonal referral teaching hospital in northern Tanzania. Located in Moshi district-Tanzania, it is the third largest hospital in the country with 700 official inpatient beds often stretched to accommodate about 100–150 more patients on canvas beds. The hospital serves the country’s northern corridor with an estimated catchment population of 15 million people per the national census data, with some patients traveling a distance of up to 800 km from their place of residence [12].

The Orthopaedic ward admission registry was reviewed for all patients admitted during the study review period. All patients aged 18 years and younger were identified by their diagnosis and further classified by name and file identification number if diagnosed with a femur fracture. Files for patients with a femur fracture were retrieved from the medical records department and were sub-categorized based on fracture location and type of fracture (pathological versus traumatic). All patients with incomplete data were excluded from the study.

A standardized data extraction form was designed to record all demographic and pertinent injury data including age and gender of the patient, admission date, injury mechanisms, site of the fracture, type of associated injury, fracture type, fracture location, treatment type and duration of hospital stay. Collected data were analysed using SPSS (Statistical Package for Social Sciences) software version 22 (IBM Corp., Armonk, NY, USA). Frequencies, percentages, mean and median were calculated and summarized by narration, frequency tables, histograms and pie charts. Multivariate analysis with a developed logistic regression with age, mechanism of injury, gender and fracture type being included based on similar studies and Fisher exact tests were computed to determine the factors for associated injuries and their corresponding 95% confidence intervals were also calculated. A *P*-value less than 0.05 was considered statistically significant.

## Results

**Fig. 1:**
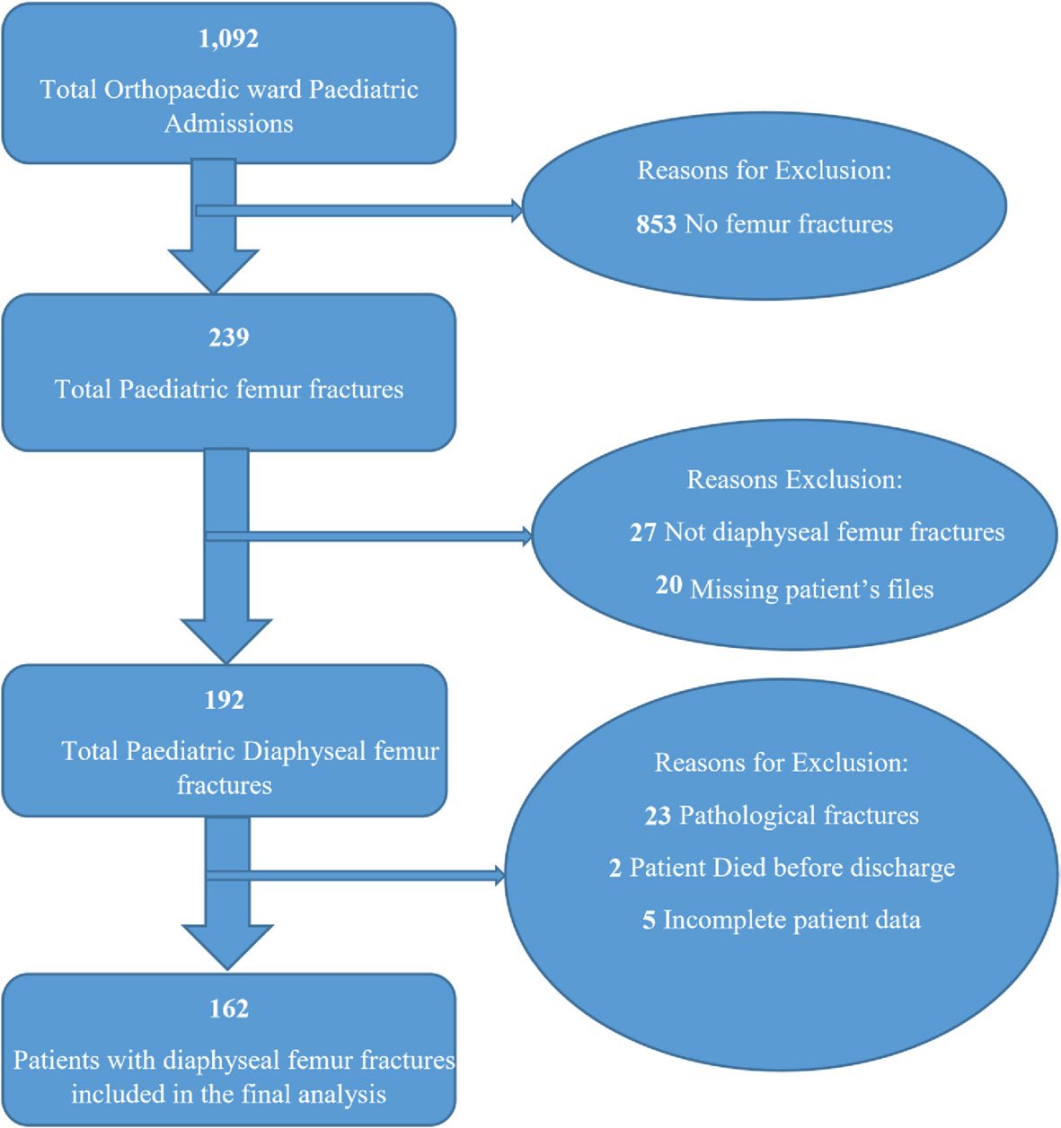
Diaphyseal femur fractures recruitment flowchart

Over the study period, a total of 1,092 paediatric patients were admitted to the orthopaedic ward of which 192 (17.6%) were diagnosed with a diaphyseal femur fracture (Fig. 1). Of this group, 162 (84.4%) diaphyseal femur fractures met the inclusion criteria to be in the final patient cohort for analysis, resulting in a diaphyseal femur fracture prevalence of 18%.

Of this cohort, the median age was 8 years IQR (4–12) and 72 patients (44.4%) were aged between 6 – 12 years. Most fractures were seen in males 111 (68.5%) with more than half of the patients being primary school children (Table 1). In addition, one-hundred forty-four (88.9%) patients had no health insurance, and 64 patients (39.5%) had fractures that occurred while along the road-side.

**Table 1:** Patient Demographics (*n* = 162)

Characteristics	N	%
Median Age in years (IQR)	8 (4–12)	
Age category in years		
< 1	3	1.9
1 – 2	21	13.0
3 – 5	31	19.1
6—12	72	44.4
13 – 18	35	21.6
Sex		
Male	111	68.5
Female	51	31.5
Level of Education		
Preschool	36	22.2
Kindergarten	22	13.6
Primary	86	53.1
Secondary	18	11.1
Insurance status		
Insured	18	11.1
Not insured	144	88.9
Location at time of injury		
Home	54	33.3
School	13	8
Road	64	39.5
Public playground	18	11.1
Other	13	8

Most femur fractures were closed 156 (96.3%). Eighty fractures (49.4%) were mid-diaphyseal femur fractures with a transverse fracture being the most common pattern. Skeletal traction was the most frequent inpatient fracture immobilization method observed. 69.1% of patients were treated Non-operatively with 104 (65.4%) having longer than two weeks length of hospital stay (Table 2).

**Table 2:** Patient Injury Characteristics (*n* = 162)

Characteristics	N	%
Fracture type		
Closed	156	96.3
Open	6	3.7
Fracture location		
Proximal	50	30.9
Middle	80	49.4
Distal	32	19.8
Side of femur fracture		
Right	85	52.5
Left	72	44.4
Both	5	3.1
Fracture pattern		
Transverse	79	48.8
Oblique	36	22.2
Spiral	33	20.4
Comminuted	14	8.6
Type of traction		
Skin	53	32.7
Skeletal	109	67.3
Treatment Type		
Non Operative	112	69.1
Operative	50	30.9
Length Of Hospital Stay		
≤ 7 Days	22	13.6
8 – 14 Days	34	21
14 >	106	65.4

All children under one-year of age sustained a diaphyseal femur fracture from a fall at heights less than two meters. Toddlers (1–2 years) had most of their fractures result from falls of less than two meters followed by falling objects as the second most common mechanism of injury. Fractures resulting from RTIs were predominantly seen in older children, the 6–12 and 13–18 years of age categories, respectively. There was a significant number of children aged within 3–5 years age group whom sustained diaphyseal femur fractures from a pedestrian versus a motorcycle accident (MCA). Pedestrian RTIs was the leading mechanism of injury for this cohort of paediatric diaphyseal femur fractures (Table 3).

**Table 3:** Mechanism of Injury of Paediatric Diaphyseal Femur Fractures by Age (*n* = 162)

Mechanism of Injury		Age Categories in Years					N	%
		< 1	1—2	3—5	6—12	13—18		
Fall								
	Running	0	6	5	4	3	18	11.1
≤ 2 m	Playground	0	2	2	8	8	20	12.3
	Furniture	3	6	3	4	0	16	9.9
	Other	0	1	5	7	3	16	9.9
> 2 m	Tree	0	0	0	16	7	23	14.2
RTI								
Pedestrian MCA		0	2	10	12	4	28	17.3
Pedestrian MVA		0	0	0	11	1	12	7.4
Pillion^a^ MCA		0	0	3	3	4	10	6.2
Passenger MVA		0	0	1	2	5	8	4.9
Falling Objects								
Gate/Doors/walls		0	1	1	2	0	4	2.5
Other objects		0	3	1	3	0	7	4.3

^a^Pillion refers to a patient was carried on a motorcycle at the time of injury

*MCA* Motorcycle Accident

*MVA* Motor vehicle Accident

**Fig. 2:**
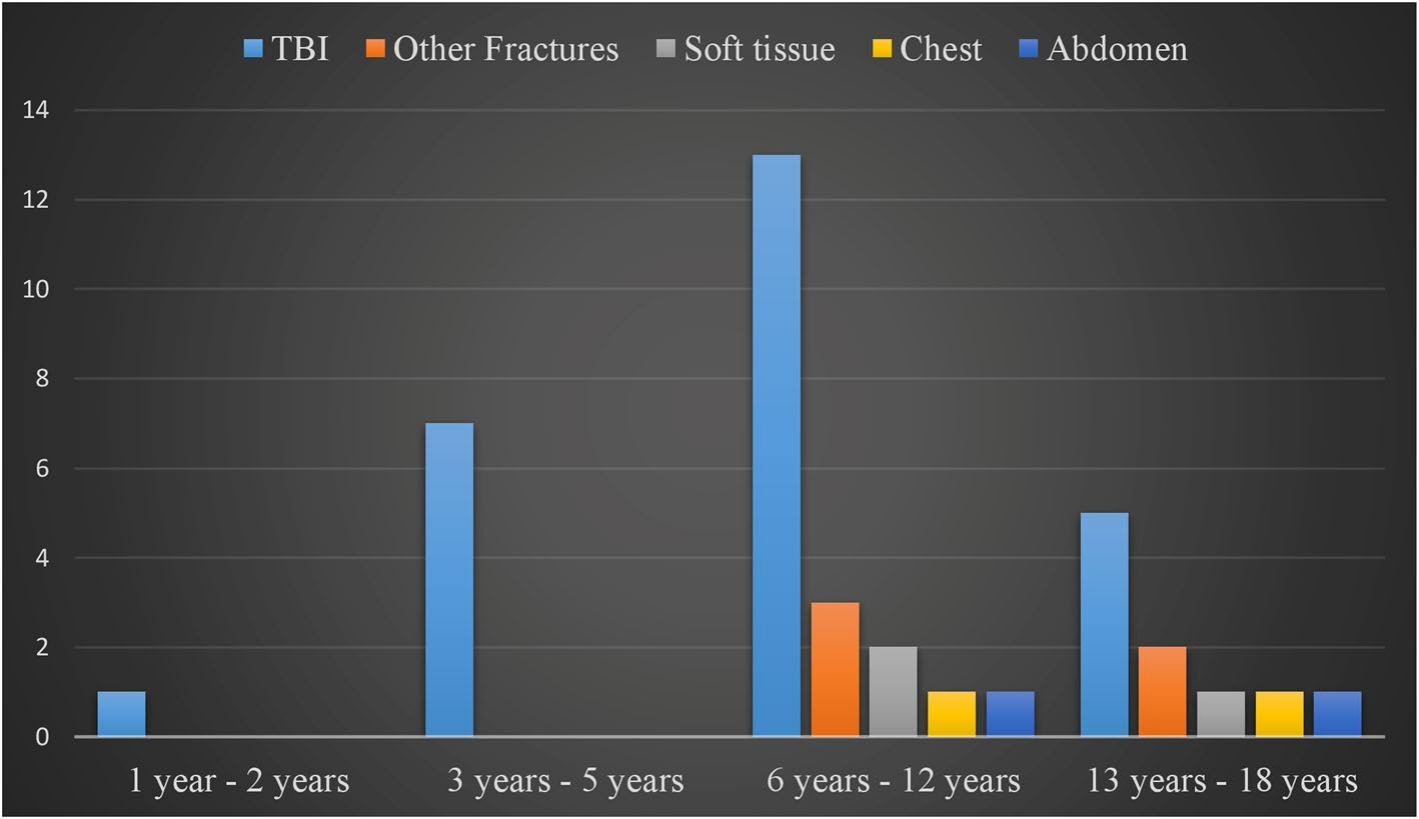
Distribution of associated injuries across different age categories (*n* = 38)

A total of 38 (23.5%) diaphyseal femur fracture patients presented with associated injuries, with traumatic brain injury (TBI) seen in 26 (69%) patients in this group; and was the most common associated injury across all age groups. Most associated injuries were seen in the 6 – 12 year-old group. Combined TBI, abdominal injury and femur fracture (Waddell’s triad) was present in three (8%) patients with associated injuries (Fig. 2).

Children in the older age categories were more likely to sustain an associated injury compared to younger children. When comparing the age categories, the 6–12 year-old and the 13–18 year–old groups had 8 and 11 times higher odds for associated injuries, respectively. Patients with an open fracture had statistically significant more associated injuries (*p* < 0.001), looking at fracture patterns those with a spiral fracture had the least associated injuries. (Table 4). Children with fractures because of a RTI exhibited 17 times higher odds of associated injuries compared to a fall.

**Table 4:** Factors related to Diaphyseal Femur Fracture Associated Injuries (*n* = 162)

Associated injuries				
	Yes	Total		
	n (%)	n (%)		
Factors	38 (23.5)	162 (100)	OR (95% CI)	*p*-value
Age (years)				
≤ 2	1 (2.6)	24 (14.8)	1	
3 to 5	7 (18.4)	31 (19.1)	6.71 (0.76—58.87)	0.086
6 to 12	19 (50.0)	72 (44.4)	8.25 (1.04—65.31)	0.046
13 to 18	11 (28.9)	35 (21.6)	10.54 (1.26—88.31)	0.031
Sex				
Male	25 (65.8)	111 (68.5)	1	
Female	13 (34.2)	51 (31.5)	1.18 (0.54—2.55)	0.679
Fracture type^a^				
Closed	32 (84.2)	156 (96.3)	-	
Open	6 (15.8)	6 (3.7)	-	< 0.001
Fracture pattern				
Transverse	23 (60.5)	79 (48.8)	1	
Oblique	8 (21.1)	36 (22.2)	0.69 (0.27—1.75)	0.441
Spiral	2 (5.3)	33 (20.4)	0.16 (0.03—0.71)	0.016
Comminuted	5 (13.2)	14 (8.6)	1.35 (0.41—4.47)	0.621
Injury Mechanism				
Falling	6 (15.8)	93 (57.4)	1	
RTI	30 (78.9)	58 (35.8)	16.73 (6.28—44.57)	< 0.001
Falling objects	2 (5.3)	11 (6.8)	2.64 (0.47—14.71)	0.269

^a^ Fisher’s exact test

*CI* Confidence Interval

*OR* Odds Ratio

*RTI* Road Traffic Injuries

## Discussion

### Prevalence

The prevalence of diaphyseal femur fractures among paediatric orthopaedic admissions in this study was 18%. This prevalence conforms to the reported Sub-Saharan prevalence observed in hospital-based studies conducted in Nigeria, Cameroon and Uganda which reported 10%, 16% and 22%, respectively [13–15].

Studies showing a lower prevalence of paediatric diaphyseal femur fractures in the literature [4, 16, 17], were population based retrospective studies in Europe and America and had a prevalence of 1.7%—5%. The large difference in patient cohort size when comparing hospital-based and population-based studies could explain this discrepancy. Our hospital-based study might underestimate the true paediatric diaphyseal femur fracture prevalence for the region as some patients are missed due to treatment at other facilities or have sought treatment with a traditional bone setter.

We also found that males were more likely to sustain a diaphyseal femur fracture, as they represented 111 (68.5%) patients in the cohort with a M:F ratio of 2.2:1. Our findings are similar to previous studies that showed male patients accounting for 62–73% of femur fracture patients and requires an aggressive preventive strategy for this subset of the population [2, 5, 18].

### Mechanism of injury

In our study, the most common injury mechanism was a fall accounting for 57.4% (93) of all fractures and nearly half of these falls were in children in their home environment. Earlier studies also demonstrated falls as a common mechanism of injury [6, 14, 15, 19]. We found that most falls were from trees (25%, 23), while other studies have documented that most occurred during playground activities [18, 19]. Regional variation in childhood recreational activities in addition to the habit of harvesting fruits and firewood from trees which is observed in our society might explain this difference.

Akinyoola et al*.* from Nigeria and Mughal et al*.*in South Africa reported RTI as the most common mechanism of injury throughout Sub-Saharan Africa [2, 8]. We found that RTIs resulting from a pedestrian versus MCA was the most common mechanism for sustaining a diaphyseal femur fracture accounting for 17.3% of overall childhood diaphyseal femur fractures. The absence of adequate infrastructure, poor traffic regulations and a lack of adult supervision most likely results in a higher risk of sustaining these injuries in the paediatric population. Child education on pedestrian safety and implementation of road safety rules is paramount to prevent a continued rise in the prevalence of fractures [13, 18].

Our data did not demonstrate the expected variation in the mechanism of injury across age groups. Other studies have shown a transition from falls to RTI as the major mechanism when shifting from younger to older children. Socio-economic activities in our region that involve tree climbing attribute the overrepresentation of falls from a height [18, 19]. In addition, 54 (33%) of the femur fractures in our study occurred at home which poses a challenge as children are considered safe while at home due to theoretical adult supervision. This necessitates a need for public health interventions to further understand the risk, design appropriate family level education and prevent these injuries in the future.

### Associated injuries

We found a 23.5% prevalence of femur fracture associated injuries that agree with other published studies, demonstrating a prevalence ranging from 28.6% in the USA to 36% in New Zealand [6, 7]. Being older than 6 years and being female increased the odds of sustaining an associated injury that is similar to findings reported in the literature [6, 7]. Older children are more likely to sustain a high energy injury mechanism, either a RTI or a fall from a height above two meters, which explains the predisposition to these associated injuries.

TBI was the most common associated injury in our patient cohort. Other studies have reported that other long-bone fractures are the most common associated injury followed by TBI. Some studies demonstrated the use of helmets and other protective equipment during contact or motorized sports to have a protective effect in high energy injuries and hence minimize the extent of bodily injuries. This is an area that could be further explored in our settings with future studies. Sports related falls were the most common mechanism of sustaining a femur fracture in these other studies, while our study saw a higher incidence of RTI with pedestrian versus MCA (17.3%) [16, 20].

Fractures resulting from RTIs had an increased risk of having an associated injury that is similar to other studies in the literature [2, 17]. RTI also increased the risk of Waddell’s triad, which was 3 (8%) of those with associated injuries in this study, similar to that seen in Nigeria (8.7%) but significantly lower that seen in the USA (25.5%) [6, 8]. Understanding this association is critical in determining patient care requirements from the injury scene all the way until definitive hospital treatment as some associated injuries are life threatening if missed.

### Limitations and strength

This study being retrospective could not control for the quality of the information found in the patient files and this resulted in 25 (13%) of diaphyseal femur fractures being excluded from the study. The study established baseline data in paediatric diaphyseal femur fractures at our centre, which will help improve the management of these injuries.

### Recommendations

Childhood diaphyseal femur fractures address a crucial component in children health and safety as these injuries often times are a result of high energy trauma as in this study pedestrian MCA and falling from trees were the top two leading specific mechanisms of injury, respectively. The need for a population-based study exists to obtain a better understanding of this phenomenon, as other children continue to be treated at different facilities or traditional bone setters. More emphasis on road safety measures for drivers and pedestrians is critical as RTI attributed to most those with associated injuries and was the second leading cause of paediatric diaphyseal femur fractures.

## Conclusion

Falls contributed to most diaphyseal femur fractures sustained in our cohort of paediatric patients, but pedestrian versus MCA was the leading mechanism of injury followed by falling from trees. The 18% diaphyseal femur fractures prevalence in our settings correlates with injury burden observed in other hospital-based studies within the region. TBI was the most common associated injury with RTI injuries contributing to the majority of them. It was established that being over the age of 6 years and being female increased the odds of having an associated injury.

The dataset supporting the conclusions of this article is included within the article and its additional files.

## Supplementary Information

**Additional file 1.**

## Data Availability

All data generated or analysed during this study are included in this published article and is attached in supplementary information files.
