# Supplementary material for: Epidemiology and associated injuries in paediatric diaphyseal femur fractures treated at a limited resource zonal referral hospital in northern Tanzania
Source: BMC Musculoskelet Disord. 2022 Apr 18;23:360. doi: 10.1186/s12891-022-05320-x (PMC9017012; doi:10.1186/s12891-022-05320-x)
Supplement: Supplementary file 1 — Additional file 1. [file 12891_2022_5320_MOESM1_ESM.pdf]

Table 5. Paediatric diaphyseal femur fracture dataset

SN	Pt_initial	DOB	District	Region	Sex	Insure	Education	Place
1	DM	19-Sep-11	MOSHI URI	KLM	0	0	1	0
2	KM	01-Jan-04	SAME	KLM	0	0	2	0
3	RW	01-Jan-00	MONDULI	MANYARA	0	0	2	2
4	HKM	01-Jan-11	SIMANJIRO	MANYARA	0	0	1	2
5	MSM	01-Jan-03	HAI	KLM	0	0	2	3
6	AAM	01-Jan-09	MOSHI URI	KLM	0	0	1	2
7	JT	01-Jan-07	SIHA	KLM	0	0	2	2
8	HM	01-Jan-10	SAME	KLM	1	0	1	2
9	EAA	01-Jan-08	MOSHI URI	KLM	0	0	2	2
10	MWT	01-Jan-09	MOSHI URI	KLM	1	1	1	1
11	BJS	01-Jan-04	LUSHOTO	TANGA	1	0	2	1
12	MLM	01-Jan-03	SAME	KLM	1	0	2	0
13	CM	01-Feb-08	MOSHI URI	KLM	0	1	2	2
14	AM	01-Jan-13	MOSHI URI	KLM	1	0	0	0
15	ZP	01-Jan-04	MOSHI URI	KLM	1	0	2	2
16	PS	01-Jan-03	MOSHI URI	KLM	0	0	2	2
17	MT	01-Jan-07	MOSHI URI	KLM	1	0	2	0
18	PN	01-Jan-08	SIHA	KLM	0	0	2	2
19	AM	01-Jan-09	MWANGA	KLM	0	0	1	3
20	HS	01-Jan-01	MOSHI URI	KLM	0	0	2	3
21	ETS	03-Jun-11	LUSHOTO	TANGA	0	0	0	0
22	EM	07-Apr-09	SAME	KLM	1	0	1	2
25	DM	01-Jan-07	SAME	KLM	0	0	2	4
26	OEM	01-Jan-02	SAME	KLM	0	0	2	2
27	RM	01-Jan-08	MOSHI URI	KLM	0	0	2	0
28	BK	14-Oct-12	SIHA	KLM	0	0	0	2
29	JSM	01-Jan-02	LUSHOTO	TANGA	0	0	2	3
30	FM	01-Jan-11	MWANGA	KLM	0	0	0	0
31	MFR	01-Jan-09	HANDENI	TANGA	0	0	2	2
32	KD	01-Jan-98	ARUSHA	ARUSHA	0	0	3	2
33	MB	########	MOROGOR	MOROGOR	0	1	0	0
34	NV	01-Jan-05	MOSHI URI	KLM	0	1	2	1
35	MN	01-Jan-13	MWANGA	KLM	1	0	1	1
36	MEW	01-Jan-00	SIMANJIRO	MANYARA	0	0	2	2
37	MHK	26-Aug-14	SIHA	KLM	1	0	0	0
38	KS	30-Mar-09	MOSHI URI	KLM	0	0	2	3
39	SG	01-Jan-04	HAI	KLM	0	0	2	2
40	FKE	01-Jan-03	MOSHI RUI	KLM	0	0	2	0
41	MHO	01-Jan-07	SAME	KLM	0	0	2	0
42	MAS	19-Dec-12	MOSHI URI	KLM	0	1	0	0
43	APM	01-Jan-00	KOROGWE	TANGA	0	1	3	2
44	MA	01-Jan-10	SAME	KLM	0	0	1	2
45	MZ	01-Jan-06	LUSHOTO	TANGA	1	0	2	2
46	MAS	21-Mar-15	SAME	KLM	1	0	0	0

47	SG	24-Jun-00	MOSHI RUI	KLM	0	0	2	0
48	NHR	01-Jan-16	SAME	KLM	0	0	0	0
49	SY	19-Jul-13	MOSHI URI	KLM	0	0	0	0
50	ME	01-Feb-08	MOSHI RUI	KLM	0	1	2	0
51	KNE	01-Jan-07	MOSHI RUI	KLM	1	0	2	2
52	MPM	07-Jun-11	MOSHI URI	KLM	1	0	1	2
53	KE	########	MOSHI RUI	KLM	0	0	0	0
54	LED	01-Jan-09	MOSHI RUI	KLM	0	0	1	0
55	LCA	01-Jan-11	MOSHI RUI	KLM	1	0	1	2
56	MJ	01-Jan-07	MOSHI URI	KLM	0	0	2	2
57	MG	01-Jan-07	SAME	KLM	0	0	2	2
58	KJ	13-Aug-04	MOSHI URI	KLM	1	0	2	0
59	MQH	14-Jul-14	MOSHI RUI	KLM	1	0	0	0
60	TMS	01-Jan-00	MOSHI RUI	KLM	1	0	3	3
61	MFL	01-Jan-14	MOSHI RUI	KLM	1	0	0	0
62	EN	05-Jun-00	MOSHI URI	KLM	0	0	2	2
63	MI	01-Jan-14	MOSHI RUI	KLM	1	0	0	0
64	MEB	01-Jan-02	HAI	KLM	0	0	3	2
65	GJYN	03-Aug-06	BABATI	MANYARA	0	0	2	1
66	MP	22-Feb-00	MOSHI RUI	KLM	0	0	3	1
67	MIT	03-Feb-00	MWANGA	KLM	0	0	3	1
68	MN	25-Sep-15	SAME	KLM	1	0	0	0
69	KD	22-Dec-11	ARUSHA	ARUSHA	0	0	1	0
70	MD	01-Jan-07	HAI	KLM	1	0	2	1
71	KW	08-Jan-00	MOSHI RUI	KLM	0	0	2	2
72	SEA	01-Jan-05	MOSHI URI	KLM	0	0	2	2
73	SO	01-Jan-05	HAI	KLM	1	0	2	2
74	MR	06-Oct-13	HAI	KLM	0	1	0	0
75	ML	01-Jan-13	SINGIDA	SINGIDA	0	0	0	4
76	GA	16-Sep-14	HANDENI	TANGA	0	0	0	0
77	MJJ	22-Sep-14	SAME	KLM	1	0	0	0
78	CD	01-Jan-07	MOSHI URI	KLM	0	0	2	2
79	MJ	25-Mar-05	MWANGA	KLM	0	0	3	2
80	MJ	01-Jan-08	HAI	KLM	0	1	2	2
81	MS	01-Jan-04	SAME	KLM	0	0	2	2
82	KCE	02-Jan-00	MOSHI RUI	KLM	1	0	3	4
83	SL	25-Jun-14	HAI	KLM	0	0	0	0
84	MJ	01-Jan-03	SIHA	KLM	0	0	3	2
85	SP	01-Jan-00	MOSHI URI	KLM	1	0	3	2
86	MF	01-Jan-07	MWANGA	KLM	0	0	2	3
87	MA	01-Jan-04	MOSHI RUI	KLM	0	0	2	0
88	MD	03-Jan-14	SAME	KLM	1	0	0	2
89	NGG	01-Jan-13	MWANGA	KLM	1	0	0	2
90	MD	20-Nov-06	SAME	KLM	0	0	2	3
91	RU	01-Jan-09	MOSHI URI	KLM	1	1	2	0
92	MSR	01-Jan-02	SIHA	KLM	0	0	2	4
93	SJA	01-Jan-02	MOSHI RUI	KLM	1	0	3	1

94	SA	01-Jan-00	LUSHOTO	TANGA	0	0	3	3
95	ML	01-Jan-00	SAME	KLM	1	0	2	2
96	MHR	01-Apr-17	SAME	KLM	0	0	0	0
97	TG	11-Jul-09	ROMBO	KLM	0	0	2	2
98	MJS	21-Aug-08	MWANGA	KLM	0	0	2	3
99	MGJ	01-Jan-10	MOSHI URI	KLM	0	0	2	2
100	SBN	17-Dec-11	SIHA	KLM	0	0	2	2
101	MIY	########	MWANGA	KLM	0	0	3	1
102	NBS	01-Jan-05	MOSHI RUI	KLM	0	0	2	4
103	MFS	10-Feb-11	SAME	KLM	1	0	2	1
104	MWR	01-Jan-11	MWANGA	KLM	1	0	2	0
105	MJP	01-Aug-02	SIHA	KLM	0	0	2	4
106	MD	22-Mar-15	MWANGA	KLM	1	0	0	2
107	MAP	01-Jan-14	MOSHI RUI	KLM	0	0	1	2
108	MC	14-Dec-13	MWANGA	KLM	0	1	0	0
109	RFF	01-Jan-08	HAI	KLM	1	0	2	1
110	SJD	01-Jan-00	MWANGA	KLM	0	0	3	3
111	MG	01-Jan-04	SAME	KLM	0	0	3	4
112	MI	01-Jan-09	MWANGA	KLM	0	0	2	0
113	MEG	24-Dec-12	LUSHOTO	TANGA	0	0	1	0
114	KN	25-Nov-15	MOSHI RUI	KLM	1	0	0	0
115	SS	21-Nov-13	SIHA	KLM	0	0	1	2
116	KRM	01-Jan-13	MWANGA	KLM	0	0	1	0
117	MOT	01-Jan-01	MOSHI URI	KLM	0	0	2	2
118	KS	01-Jan-09	MOSHI URI	KLM	0	1	2	0
119	MRH	12-Aug-12	MOSHI URI	KLM	0	0	2	2
120	MEL	23-Jun-06	HAI	KLM	1	0	2	0
121	LTT	11-Sep-16	MANYARA	KLM	0	0	0	0
122	AE	01-Jan-04	MOSHI URI	KLM	0	0	2	2
123	CJE	01-Jan-06	HANDENI	TANGA	0	0	2	4
124	KF	01-Jan-09	ARUMERU	ARUSHA	0	0	2	3
125	MSE	01-Jan-07	SAME	KLM	0	0	2	0
126	MAC	14-Apr-05	MOSHI RUI	KLM	0	0	2	1
127	MSS	04-Aug-13	MOSHI URI	KLM	0	0	0	0
128	GJY	04-Aug-06	BABATI	MANYARA	0	0	2	0
129	TUJ	18-Apr-10	MOSHI RUI	KLM	1	0	2	2
130	MEJ	01-Jan-08	MOSHI RUI	KLM	0	0	2	4
131	MM	01-Jan-00	SAME	KLM	0	0	3	2
132	KAA	01-Jan-04	MWANGA	KLM	0	0	2	0
133	SP	########	MOSHI RUI	KLM	0	0	2	2
134	SM	01-Jan-11	SIHA	KLM	1	0	1	4
135	MB	01-Jan-10	MOSHI RUI	KLM	0	0	2	2
136	TD	01-Jan-03	MWANGA	KLM	1	0	2	2
137	TAG	01-Jan-01	MOSHI RUI	KLM	0	0	2	3
138	NBJ	01-Jan-13	ARUMERU	KLM	1	0	0	0
139	MV	01-Jan-09	MOSHI RUI	KLM	0	0	2	2
140	MH	01-Jan-08	MOSHI URI	KLM	0	0	2	2

141	SNM	01-Jan-01	MOSHI URI	KLM	1	0	2	2
142	MCK	23-Jul-11	SIMANJIRO	MANYARA	1	1	1	2
143	MCO	01-Jul-09	MOSHI URI	KLM	0	0	2	0
144	MGW	26-Feb-14	MOSHI URI	KLM	1	0	0	4
145	MF	01-Jan-08	MOSHI RUI	KLM	0	0	2	2
146	ML	01-Jan-03	SAME	KLM	1	0	2	4
147	MAJ	01-Jan-10	MOSHI RUI	KLM	0	0	1	2
148	NN	14-Sep-13	MOSHI URI	KLM	1	0	0	0
149	MD	01-Jan-11	MOSHI URI	KLM	0	1	1	0
150	KM	01-Jan-12	MOSHI URI	KLM	0	0	0	0
151	TJ	01-Jan-11	LUSHOTO	TANGA	0	1	0	2
152	LJ	01-Jan-06	MOSHI URI	KLM	1	0	2	2
153	ME	01-Jan-11	MOSHI URI	KLM	0	0	0	3
154	MB	01-Jan-08	LUSHOTO	TANGA	1	0	2	3
155	MB	01-Jan-01	MOSHI RUI	KLM	0	0	3	2
156	KS	01-Jan-06	ARUSHA	ARUSHA	0	0	2	4
157	SS	01-Jan-00	MOSHI URI	KLM	0	0	2	2
158	MR	01-Jan-00	MOSHI URI	KLM	1	1	3	3
159	MS	01-Jan-05	LUSHOTO	TANGA	0	0	1	0
160	NY	01-Jan-14	MOSHI RUI	KLM	0	0	0	0
161	LT	01-Jan-04	MOSHI RUI	KLM	0	0	2	2
162	LG	01-Jan-06	SIMANJIRO	ARUSHA	1	1	2	0
163	BM	01-Jan-08	MOSHI URI	KLM	1	0	2	3
164	MH	01-Jan-13	MOSHI URI	KLM	0	1	0	3

Place_Othe	Femur	Mechanism	Mech_fall	Mech_fall_	Mech_fall_	MTC	Assault	Assau_Othe
	1	1	0	4	HANGING F	#NULL!	#NULL!	
	1	3	#NULL!	#NULL!		#NULL!	#NULL!	
	0	2	#NULL!	#NULL!		2	#NULL!	
	1	2	#NULL!	#NULL!		0	#NULL!	
	0	1	1	0		#NULL!	#NULL!	
	0	2	#NULL!	#NULL!		2	#NULL!	
	1	2	#NULL!	#NULL!		2	#NULL!	
	1	2	#NULL!	#NULL!		0	#NULL!	
	1	2	#NULL!	#NULL!		1	#NULL!	
	0	1	0	1		#NULL!	#NULL!	
	1	1	0	1		#NULL!	#NULL!	
	1	1	0	1		#NULL!	#NULL!	
	1	2	#NULL!	#NULL!		1	#NULL!	
	1	1	0	4	BROTHER D	#NULL!	#NULL!	
	0	2	#NULL!	#NULL!		2	#NULL!	
	0	2	#NULL!	#NULL!		3	#NULL!	
	0	1	0	0		#NULL!	#NULL!	
	0	2	#NULL!	#NULL!		1	#NULL!	
	1	1	0	1		#NULL!	#NULL!	
	1	1	0	1		#NULL!	#NULL!	
	0	1	0	3		#NULL!	#NULL!	
	0	1	0	4	TRIPED GO	#NULL!	#NULL!	
FARM	2	3	#NULL!	#NULL!		#NULL!	#NULL!	
	1	1	0	4	FELL INTO A	#NULL!	#NULL!	
	1	1	0	4	FELL FROM	#NULL!	#NULL!	
	1	2	#NULL!	#NULL!		0	#NULL!	
	1	1	0	1		#NULL!	#NULL!	
	0	1	0	1		#NULL!	#NULL!	
	0	2	#NULL!	#NULL!		1	#NULL!	
	1	2	#NULL!	#NULL!		3	#NULL!	
	0	1	0	3		#NULL!	#NULL!	
	0	1	0	2		#NULL!	#NULL!	
	0	3	#NULL!	#NULL!		#NULL!	#NULL!	
	1	1	0	4	FROM A BI	#NULL!	#NULL!	
	1	3	#NULL!	#NULL!		#NULL!	#NULL!	
	1	1	0	2		#NULL!	#NULL!	
	1	2	#NULL!	#NULL!		0	#NULL!	
	0	1	0	1		#NULL!	#NULL!	
	0	1	1	0		#NULL!	#NULL!	
	1	2	#NULL!	#NULL!		0	#NULL!	
	1	2	#NULL!	#NULL!		3	#NULL!	
	0	2	#NULL!	#NULL!		1	#NULL!	
	1	2	#NULL!	#NULL!		0	#NULL!	
	0	1	0	3		#NULL!	#NULL!	

	0	1	1	0		#NULL!	#NULL!
	0	1	0	3		#NULL!	#NULL!
	0	3	#NULL!	#NULL!		#NULL!	#NULL!
	0	1	1	0		#NULL!	#NULL!
	1	2	#NULL!	#NULL!		0	#NULL!
	1	2	#NULL!	#NULL!		0	#NULL!
	0	1	0	3		#NULL!	#NULL!
	0	3	#NULL!	#NULL!		#NULL!	#NULL!
	1	2	#NULL!	#NULL!		0	#NULL!
	1	1	0	4	BICYCLE	#NULL!	#NULL!
	2	2	#NULL!	#NULL!		0	#NULL!
	0	1	0	3		#NULL!	#NULL!
	1	3	#NULL!	#NULL!		#NULL!	#NULL!
	0	1	0	2		#NULL!	#NULL!
	1	1	0	2		#NULL!	#NULL!
	2	1	1	0		#NULL!	#NULL!
	0	1	0	2		#NULL!	#NULL!
	1	2	#NULL!	#NULL!		2	#NULL!
	1	1	0	2		#NULL!	#NULL!
	0	1	0	2		#NULL!	#NULL!
	0	1	0	2		#NULL!	#NULL!
	0	1	0	1		#NULL!	#NULL!
	0	1	0	1		#NULL!	#NULL!
	1	1	1	0		#NULL!	#NULL!
	0	2	#NULL!	#NULL!		3	#NULL!
	0	2	#NULL!	#NULL!		0	#NULL!
	0	2	#NULL!	#NULL!		0	#NULL!
	0	1	0	1		#NULL!	#NULL!
FARM	1	1	0	4	FARM PIT	#NULL!	#NULL!
	1	1	0	1		#NULL!	#NULL!
	0	3	#NULL!	#NULL!		#NULL!	#NULL!
	1	1	1	0		#NULL!	#NULL!
	0	2	#NULL!	#NULL!		0	#NULL!
	2	2	#NULL!	#NULL!		1	#NULL!
	0	2	#NULL!	#NULL!		0	#NULL!
FARM	1	1	0	4	FARM TRIP	#NULL!	#NULL!
	0	1	0	3		#NULL!	#NULL!
	0	2	#NULL!	#NULL!		2	#NULL!
	0	2	#NULL!	#NULL!		3	#NULL!
	1	1	1	0		#NULL!	#NULL!
	0	1	1	0		#NULL!	#NULL!
	0	2	#NULL!	#NULL!		0	#NULL!
	0	2	#NULL!	#NULL!		0	#NULL!
	0	1	1	0		#NULL!	#NULL!
	1	1	0	3		#NULL!	#NULL!
FARM	0	1	1	0		#NULL!	#NULL!
	0	1	0	2		#NULL!	#NULL!

	0	1	0	2		#NULL!	#NULL!
	1	2	#NULL!	#NULL!		0	#NULL!
	1	1	0	1		#NULL!	#NULL!
	0	2	#NULL!	#NULL!		3	#NULL!
	0	3	#NULL!	#NULL!		#NULL!	#NULL!
	0	2	#NULL!	#NULL!		1	#NULL!
	1	2	#NULL!	#NULL!		0	#NULL!
	0	1	0	2		#NULL!	#NULL!
FARM	0	1	1	0		#NULL!	#NULL!
	0	1	0	1		#NULL!	#NULL!
	1	1	1	0		#NULL!	#NULL!
FARM	1	1	0	4	KNOCKED E	#NULL!	#NULL!
	1	2	#NULL!	#NULL!		0	#NULL!
	1	2	#NULL!	#NULL!		2	#NULL!
	0	3	#NULL!	#NULL!		#NULL!	#NULL!
	0	1	0	2		#NULL!	#NULL!
	0	1	0	2		#NULL!	#NULL!
FARM	2	1	1	0		#NULL!	#NULL!
	0	1	0	3		#NULL!	#NULL!
	0	1	0	3		#NULL!	#NULL!
	1	1	0	3		#NULL!	#NULL!
	0	2	#NULL!	#NULL!		0	#NULL!
	1	1	0	2		#NULL!	#NULL!
	0	2	#NULL!	#NULL!		0	#NULL!
	0	3	#NULL!	#NULL!		#NULL!	#NULL!
	1	2	#NULL!	#NULL!		0	#NULL!
	0	1	0	2		#NULL!	#NULL!
	0	1	0	3		#NULL!	#NULL!
	1	2	#NULL!	#NULL!		1	#NULL!
FARM	0	1	0	4	FELL INTO I	#NULL!	#NULL!
	1	1	1	0		#NULL!	#NULL!
	1	1	1	0		#NULL!	#NULL!
	1	1	0	2		#NULL!	#NULL!
	1	1	0	1		#NULL!	#NULL!
	1	1	0	4	FELL INTO I	#NULL!	#NULL!
	1	2	#NULL!	#NULL!		0	#NULL!
FARM	0	1	1	0		#NULL!	#NULL!
	0	2	#NULL!	#NULL!		2	#NULL!
	1	1	1	0		#NULL!	#NULL!
	1	2	#NULL!	#NULL!		2	#NULL!
FARM	1	1	1	0		#NULL!	#NULL!
	0	2	#NULL!	#NULL!		1	#NULL!
	1	2	#NULL!	#NULL!		0	#NULL!
	0	1	0	1		#NULL!	#NULL!
	0	1	0	3		#NULL!	#NULL!
	1	2	#NULL!	#NULL!		1	#NULL!
	1	2	#NULL!	#NULL!		0	#NULL!

	1	2	#NULL!	#NULL!		0	#NULL!
	0	2	#NULL!	#NULL!		3	#NULL!
	0	1	1	0		#NULL!	#NULL!
CHURCH	1	1	0	1		#NULL!	#NULL!
	0	2	#NULL!	#NULL!		1	#NULL!
FARM	1	1	1	0		#NULL!	#NULL!
	1	2	#NULL!	#NULL!		0	#NULL!
	1	1	0	3		#NULL!	#NULL!
	0	1	0	4	HANGING F	#NULL!	#NULL!
	0	1	0	1		#NULL!	#NULL!
	1	2	#NULL!	#NULL!		2	#NULL!
	0	2	#NULL!	#NULL!		0	#NULL!
	0	1	0	4	DITCH	#NULL!	#NULL!
	1	1	0	2		#NULL!	#NULL!
	0	2	#NULL!	#NULL!		3	#NULL!
FARM	0	1	0	4	FELL INTO	#NULL!	#NULL!
	0	2	#NULL!	#NULL!		1	#NULL!
	0	1	0	2		#NULL!	#NULL!
	1	1	1	0		#NULL!	#NULL!
	1	1	0	3		#NULL!	#NULL!
	0	1	0	4	TRENCH	#NULL!	#NULL!
	1	1	0	3		#NULL!	#NULL!
	0	1	0	2		#NULL!	#NULL!
	0	1	0	2		#NULL!	#NULL!

Falling_Obj	Objects_Ot	Other_caus	Fract_Type	Fract_Loca	Fract_Patte	Both_Lt_Ty	Both_Lt_Lo	Both_Lt_Pa
#NULL!			0	1	2	#NULL!	#NULL!	#NULL!
4	His friend fell on him		0	0	2	#NULL!	#NULL!	#NULL!
#NULL!			0	0	0	#NULL!	#NULL!	#NULL!
#NULL!			0	1	1	#NULL!	#NULL!	#NULL!
#NULL!			0	2	3	#NULL!	#NULL!	#NULL!
#NULL!			0	1	0	#NULL!	#NULL!	#NULL!
#NULL!			0	1	0	#NULL!	#NULL!	#NULL!
#NULL!			0	0	1	#NULL!	#NULL!	#NULL!
#NULL!			0	0	0	#NULL!	#NULL!	#NULL!
#NULL!			0	0	2	#NULL!	#NULL!	#NULL!
#NULL!			0	1	1	#NULL!	#NULL!	#NULL!
#NULL!			0	2	1	#NULL!	#NULL!	#NULL!
#NULL!			0	1	0	#NULL!	#NULL!	#NULL!
#NULL!			0	1	2	#NULL!	#NULL!	#NULL!
#NULL!			1	2	2	#NULL!	#NULL!	#NULL!
#NULL!			0	2	0	#NULL!	#NULL!	#NULL!
#NULL!			0	2	1	#NULL!	#NULL!	#NULL!
#NULL!			0	2	1	#NULL!	#NULL!	#NULL!
#NULL!			0	0	1	#NULL!	#NULL!	#NULL!
#NULL!			0	2	0	#NULL!	#NULL!	#NULL!
#NULL!			0	1	1	#NULL!	#NULL!	#NULL!
#NULL!			0	1	2	#NULL!	#NULL!	#NULL!
2			0	0	0	0	1	1
#NULL!			0	2	0	#NULL!	#NULL!	#NULL!
#NULL!			0	1	2	#NULL!	#NULL!	#NULL!
#NULL!			0	1	1	#NULL!	#NULL!	#NULL!
#NULL!			0	0	0	#NULL!	#NULL!	#NULL!
#NULL!			0	1	0	#NULL!	#NULL!	#NULL!
#NULL!			0	0	0	#NULL!	#NULL!	#NULL!
#NULL!			0	2	3	#NULL!	#NULL!	#NULL!
#NULL!			0	1	2	#NULL!	#NULL!	#NULL!
#NULL!			0	2	3	#NULL!	#NULL!	#NULL!
4	FELLOW STUDENT FELI		0	1	2	#NULL!	#NULL!	#NULL!
#NULL!			0	0	0	#NULL!	#NULL!	#NULL!
4	BRICK PIECE		0	1	1	#NULL!	#NULL!	#NULL!
#NULL!			0	1	2	#NULL!	#NULL!	#NULL!
#NULL!			0	1	0	#NULL!	#NULL!	#NULL!
#NULL!			0	0	0	#NULL!	#NULL!	#NULL!
#NULL!			0	0	1	#NULL!	#NULL!	#NULL!
#NULL!			0	1	0	#NULL!	#NULL!	#NULL!
#NULL!			1	1	0	#NULL!	#NULL!	#NULL!
#NULL!			0	1	0	#NULL!	#NULL!	#NULL!
#NULL!			0	2	0	#NULL!	#NULL!	#NULL!
#NULL!			0	1	1	#NULL!	#NULL!	#NULL!

#NULL!		0	1	0	#NULL!	#NULL!	#NULL!
#NULL!		0	1	0	#NULL!	#NULL!	#NULL!
4	BICYCLE	0	1	1	#NULL!	#NULL!	#NULL!
#NULL!		0	1	0	#NULL!	#NULL!	#NULL!
#NULL!		1	2	0	#NULL!	#NULL!	#NULL!
#NULL!		0	1	0	#NULL!	#NULL!	#NULL!
#NULL!		0	0	0	#NULL!	#NULL!	#NULL!
4	MAIZE SUC	0	0	2	#NULL!	#NULL!	#NULL!
#NULL!		0	0	2	#NULL!	#NULL!	#NULL!
#NULL!		0	0	1	#NULL!	#NULL!	#NULL!
#NULL!		0	1	1	0	1	0
#NULL!		0	2	0	#NULL!	#NULL!	#NULL!
0		0	1	2	#NULL!	#NULL!	#NULL!
#NULL!		0	1	0	#NULL!	#NULL!	#NULL!
#NULL!		0	1	2	#NULL!	#NULL!	#NULL!
#NULL!		0	0	0	1	0	3
#NULL!		0	0	1	#NULL!	#NULL!	#NULL!
#NULL!		0	1	3	#NULL!	#NULL!	#NULL!
#NULL!		0	0	2	#NULL!	#NULL!	#NULL!
#NULL!		0	2	2	#NULL!	#NULL!	#NULL!
#NULL!		0	2	1	#NULL!	#NULL!	#NULL!
#NULL!		0	0	0	#NULL!	#NULL!	#NULL!
#NULL!		0	0	0	#NULL!	#NULL!	#NULL!
#NULL!		0	0	0	#NULL!	#NULL!	#NULL!
#NULL!		0	2	0	#NULL!	#NULL!	#NULL!
#NULL!		0	2	2	#NULL!	#NULL!	#NULL!
#NULL!		0	0	2	#NULL!	#NULL!	#NULL!
#NULL!		0	2	2	#NULL!	#NULL!	#NULL!
#NULL!		0	0	1	#NULL!	#NULL!	#NULL!
#NULL!		0	0	1	#NULL!	#NULL!	#NULL!
4	PARKED MOTORBIKE	0	1	2	#NULL!	#NULL!	#NULL!
#NULL!		0	0	0	#NULL!	#NULL!	#NULL!
#NULL!		0	0	3	#NULL!	#NULL!	#NULL!
#NULL!		0	0	0	1	2	1
#NULL!		0	0	3	#NULL!	#NULL!	#NULL!
#NULL!		0	1	3	#NULL!	#NULL!	#NULL!
#NULL!		0	1	2	#NULL!	#NULL!	#NULL!
#NULL!		0	1	0	#NULL!	#NULL!	#NULL!
#NULL!		0	1	0	#NULL!	#NULL!	#NULL!
#NULL!		0	1	0	#NULL!	#NULL!	#NULL!
#NULL!		0	1	2	#NULL!	#NULL!	#NULL!
#NULL!		0	1	0	#NULL!	#NULL!	#NULL!
#NULL!		0	1	1	#NULL!	#NULL!	#NULL!
#NULL!		0	0	1	#NULL!	#NULL!	#NULL!
#NULL!		0	1	0	#NULL!	#NULL!	#NULL!
#NULL!		0	2	0	#NULL!	#NULL!	#NULL!
#NULL!		0	2	2	#NULL!	#NULL!	#NULL!

#NULL!		0	1	2	#NULL!	#NULL!	#NULL!
#NULL!		0	1	0	#NULL!	#NULL!	#NULL!
#NULL!		0	2	2	#NULL!	#NULL!	#NULL!
#NULL!		0	0	0	#NULL!	#NULL!	#NULL!
4	FRIEND FELL ON HIM D	0	2	1	#NULL!	#NULL!	#NULL!
#NULL!		0	1	0	#NULL!	#NULL!	#NULL!
#NULL!		0	1	0	#NULL!	#NULL!	#NULL!
#NULL!		0	1	0	#NULL!	#NULL!	#NULL!
#NULL!		1	2	1	#NULL!	#NULL!	#NULL!
#NULL!		0	1	1	#NULL!	#NULL!	#NULL!
#NULL!		0	0	0	#NULL!	#NULL!	#NULL!
#NULL!		0	0	3	#NULL!	#NULL!	#NULL!
#NULL!		0	2	0	#NULL!	#NULL!	#NULL!
#NULL!		0	0	0	#NULL!	#NULL!	#NULL!
3		0	1	3	#NULL!	#NULL!	#NULL!
#NULL!		0	0	3	#NULL!	#NULL!	#NULL!
#NULL!		0	1	0	#NULL!	#NULL!	#NULL!
#NULL!		1	1	3	0	1	0
#NULL!		0	1	0	#NULL!	#NULL!	#NULL!
#NULL!		0	1	2	#NULL!	#NULL!	#NULL!
#NULL!		0	1	2	#NULL!	#NULL!	#NULL!
#NULL!		0	1	0	#NULL!	#NULL!	#NULL!
#NULL!		0	1	0	#NULL!	#NULL!	#NULL!
#NULL!		0	0	3	#NULL!	#NULL!	#NULL!
1		0	2	0	#NULL!	#NULL!	#NULL!
#NULL!		0	0	0	#NULL!	#NULL!	#NULL!
#NULL!		0	2	1	#NULL!	#NULL!	#NULL!
#NULL!		0	1	0	#NULL!	#NULL!	#NULL!
#NULL!		0	0	1	#NULL!	#NULL!	#NULL!
#NULL!		0	0	1	#NULL!	#NULL!	#NULL!
#NULL!		0	1	1	#NULL!	#NULL!	#NULL!
#NULL!		0	1	0	#NULL!	#NULL!	#NULL!
#NULL!		0	0	0	#NULL!	#NULL!	#NULL!
#NULL!		0	1	0	#NULL!	#NULL!	#NULL!
#NULL!		0	0	0	#NULL!	#NULL!	#NULL!
#NULL!		0	2	3	#NULL!	#NULL!	#NULL!
#NULL!		0	1	0	#NULL!	#NULL!	#NULL!
#NULL!		0	1	0	#NULL!	#NULL!	#NULL!
#NULL!		0	1	0	#NULL!	#NULL!	#NULL!
#NULL!		0	1	0	#NULL!	#NULL!	#NULL!
#NULL!		0	1	1	#NULL!	#NULL!	#NULL!
#NULL!		0	0	0	#NULL!	#NULL!	#NULL!
#NULL!		0	1	0	#NULL!	#NULL!	#NULL!
#NULL!		0	1	0	#NULL!	#NULL!	#NULL!
#NULL!		0	1	2	#NULL!	#NULL!	#NULL!
#NULL!		0	0	0	#NULL!	#NULL!	#NULL!
#NULL!		0	1	0	#NULL!	#NULL!	#NULL!

#NULL!	1	2	3	#NULL!	#NULL!	#NULL!
#NULL!	0	2	0	#NULL!	#NULL!	#NULL!
#NULL!	0	0	0	#NULL!	#NULL!	#NULL!
#NULL!	0	1	0	#NULL!	#NULL!	#NULL!
#NULL!	0	0	1	#NULL!	#NULL!	#NULL!
#NULL!	0	0	0	#NULL!	#NULL!	#NULL!
#NULL!	0	1	0	#NULL!	#NULL!	#NULL!
#NULL!	0	0	2	#NULL!	#NULL!	#NULL!
#NULL!	0	1	2	#NULL!	#NULL!	#NULL!
#NULL!	0	1	2	#NULL!	#NULL!	#NULL!
#NULL!	0	1	0	#NULL!	#NULL!	#NULL!
#NULL!	0	1	1	#NULL!	#NULL!	#NULL!
#NULL!	0	1	2	#NULL!	#NULL!	#NULL!
#NULL!	0	0	1	#NULL!	#NULL!	#NULL!
#NULL!	0	2	0	#NULL!	#NULL!	#NULL!
#NULL!	0	1	0	#NULL!	#NULL!	#NULL!
#NULL!	0	1	1	#NULL!	#NULL!	#NULL!
#NULL!	0	1	0	#NULL!	#NULL!	#NULL!
#NULL!	0	2	1	#NULL!	#NULL!	#NULL!
#NULL!	0	0	1	#NULL!	#NULL!	#NULL!
#NULL!	0	2	0	#NULL!	#NULL!	#NULL!
#NULL!	0	1	1	#NULL!	#NULL!	#NULL!
#NULL!	0	0	0	#NULL!	#NULL!	#NULL!
#NULL!	0	1	2	#NULL!	#NULL!	#NULL!

Ass_Injury	Ass_Other	Treatment	Technique_	Tech_Non_	Traction_Type
0		1	#NULL!	1	0
0		1	#NULL!	1	1
2		0	1	#NULL!	1
0		1	#NULL!	1	0
0		1	#NULL!	1	1
2		1	#NULL!	1	1
6	CLOSED PS	1	#NULL!	1	1
2		1	#NULL!	1	0
2		1	#NULL!	1	0
0		1	#NULL!	0	0
0		1	#NULL!	1	1
0		1	#NULL!	1	1
0		1	#NULL!	1	1
0		1	#NULL!	1	0
6	PATELLA F	0	6	#NULL!	#NULL!
0		1	#NULL!	1	1
0		1	#NULL!	1	1
1		1	#NULL!	1	1
0		1	#NULL!	1	1
0		1	#NULL!	1	1
0		1	#NULL!	1	0
0		1	#NULL!	1	1
0		1	#NULL!	1	1
0		1	#NULL!	1	0
0		1	#NULL!	1	1
0		1	#NULL!	1	0
0		1	#NULL!	1	1
0		1	#NULL!	1	0
0		0	2	#NULL!	0
1		0	1	#NULL!	0
0		0	0	#NULL!	0
0		1	#NULL!	2	1
0		1	#NULL!	1	0
0		0	1	#NULL!	1
0		1	#NULL!	1	0
0		1	#NULL!	1	1
2		0	2	#NULL!	1
0		0	1	#NULL!	1
0		1	#NULL!	1	1
0		1	#NULL!	1	0
6	FACE LACEI	0	1	#NULL!	1
0		0	2	#NULL!	1
0		1	#NULL!	1	1
0		1	#NULL!	2	0

0		0		2	#NULL!		1
0		1	#NULL!			1	0
0		1	#NULL!			1	0
0		0		2	#NULL!		1
6	KNEE MCL	0		6	#NULL!		1
2		1	#NULL!			1	1
0		1	#NULL!			1	1
0		1	#NULL!			1	1
0		1	#NULL!			1	0
0		1	#NULL!			0	1
0		1	#NULL!			1	1
0		1	#NULL!			2	0
0		1	#NULL!			1	0
0		0		1	#NULL!		1
0		1	#NULL!			1	0
2		0		1	#NULL!		1
0		1	#NULL!			1	1
0		1	#NULL!			0	1
0		0		2	#NULL!		1
0		0		0	#NULL!		0
0		0		0	#NULL!		1
0		1	#NULL!			1	0
0		0		2	#NULL!		0
6	R/U FRACT	1	#NULL!			0	1
2		1	#NULL!			1	1
2		0		2	#NULL!		1
0		0		2	#NULL!		1
0		1	#NULL!			1	0
0		0		0	#NULL!		0
0		1	#NULL!			1	0
0		1	#NULL!			1	0
0		1	#NULL!			1	1
2		0		2	#NULL!		1
2		1	#NULL!			0	1
0		0		2	#NULL!		1
0		0		2	#NULL!		1
0		1	#NULL!			1	0
0		0		2	#NULL!		1
6	R/U FRACT	1	#NULL!			0	1
0		0		2	#NULL!		1
0		0		2	#NULL!		1
2		1	#NULL!			1	0
2		1	#NULL!			1	0
0		0		2	#NULL!		1
0		1	#NULL!			1	0
6	CLAVICLE	1	#NULL!			2	0
0		0		1	#NULL!		1

0	0	2	#NULL!	0
0	0	1	#NULL!	1
0	1	#NULL!	2	0
2	1	#NULL!	1	1
0	1	#NULL!	1	1
0	0	2	#NULL!	1
2	1	#NULL!	1	1
0	0	1	#NULL!	1
2	0	6	#NULL!	1
0	1	#NULL!	1	0
0	1	#NULL!	1	0
0	1	#NULL!	0	1
2	1	#NULL!	1	1
0	1	#NULL!	1	0
0	1	#NULL!	1	1
0	1	#NULL!	0	1
0	0	1	#NULL!	1
2	1	#NULL!	0	1
0	0	2	#NULL!	1
0	1	#NULL!	1	1
0	1	#NULL!	1	0
0	1	#NULL!	1	1
0	1	#NULL!	1	0
0	1	#NULL!	0	1
0	0	2	#NULL!	0
0	1	#NULL!	1	1
0	0	0	#NULL!	1
0	1	#NULL!	1	1
2	0	0	#NULL!	1
0	1	#NULL!	0	1
0	0	0	#NULL!	0
0	0	0	#NULL!	1
0	1	#NULL!	1	1
0	1	#NULL!	1	0
0	0	2	#NULL!	1
2	1	#NULL!	1	1
0	0	2	#NULL!	1
0	1	#NULL!	0	1
0	0	2	#NULL!	1
0	0	2	#NULL!	1
2	1	#NULL!	1	1
4	1	#NULL!	0	1
2	1	#NULL!	0	1
0	0	2	#NULL!	1
0	1	#NULL!	1	0
2	0	2	#NULL!	1
0	1	#NULL!	1	1

6	SCALP LACI	0		6	#NULL!		1
0		1	#NULL!			1	1
0		1	#NULL!			1	1
0		1	#NULL!			1	1
2		0		2	#NULL!		1
0		1	#NULL!			0	1
2		1	#NULL!			1	0
0		1	#NULL!			2	0
0		1	#NULL!			1	0
0		1	#NULL!			1	1
2		1	#NULL!			1	1
0		1	#NULL!			1	0
0		1	#NULL!			1	1
0		1	#NULL!			1	1
0		1	#NULL!			1	1
0		1	#NULL!			1	1
4		1	#NULL!			1	1
0		1	#NULL!			1	1
0		1	#NULL!			1	1
0		1	#NULL!			2	0
0		1	#NULL!			1	0
0		1	#NULL!			1	0
0		1	#NULL!			1	1
0		1	#NULL!			1	1
